# Developing fast enzyme recycling strategy through elucidating enzyme adsorption kinetics on alkali and acid pretreated corn stover

**DOI:** 10.1186/s13068-018-1315-5

**Published:** 2018-11-20

**Authors:** Ye Yuan, Rui Zhai, Ying Li, Xiangxue Chen, Mingjie Jin

**Affiliations:** School of Environmental and Biological Engineering, Nanjing University of Science and Technology, 200 Xiaolingwei Street, Nanjing, 210094 China

**Keywords:** Enzyme recycling, Enzymatic hydrolysis, Enzyme adsorption, Lignin

## Abstract

**Background:**

Although various pre-treatment methods have been developed to disrupt the structure of lignocellulosic biomass, high dosage of cellulases is still required to hydrolyze lignocellulose to fermentable sugars. Enzyme recycling via recycling unhydrolyzed solids after enzymatic hydrolysis is a promising strategy to reduce enzyme loading for production of cellulosic ethanol.

**Results:**

To develop effective enzyme recycling method via recycling unhydrolyzed solids, this work investigated both enzymatic hydrolysis kinetics and enzyme adsorption kinetics on dilute acid and dilute alkali pre-treated corn stover (CS). It was found that most of the hydrolysable biomass was hydrolyzed in the first 24 h and about 40% and 55% of the enzymes were adsorbed on unhydrolyzed solids for dilute alkali-CS and dilute acid-CS, respectively, at 24 h of enzymatic hydrolysis. Lignin played a significant role in such adsorption and lignin materials derived from dilute acid-CS and dilute alkali-CS possessed different enzyme adsorption properties. Enzyme recycling was performed by recycling unhydrolyzed solids after 24 h enzymatic hydrolysis for five successive rounds, and successfully reduced 40% and 50% of the enzyme loadings for hydrolysis of dilute alkali-CS and for hydrolysis of dilute acid-CS, respectively.

**Conclusions:**

This study presents that the enzymes adsorbed on the unhydrolyzed solids after short-time hydrolysis could be recycled effectively for efficient enzymatic hydrolysis. Lignin derived from dilute acid-CS has higher enzyme adsorption capacity than the lignin derived from dilute alkali-CS, which led to more enzymes recycled. By applying the enzyme recycling strategy developed in this study, the enzyme dosage needed for effective cellulose hydrolysis can be significantly reduced.

## Background

Lignocellulosic biomass (including agricultural and forestry residues, municipal wastes and energy crops) is abundant, renewable, and of low cost [1, 2]. Utilization of lignocellulosic biomass for production of fuels and chemicals has attracted much attention [3], and extensive studies have been performed to develop biorefinery technologies [3–5]. One major barrier for commercialization of biorefinery technologies related to the biochemical route is the high cost of lignocellulose degrading enzymes (cellulases and hemicellulases) [6]. Lignocellulose, evolved to resist degradation by microbial organisms and enzymes, is highly recalcitrant [3, 7]. Although various pre-treatment methods have been developed to disrupt the structure of lignocellulosic biomass and to change physical and chemical properties of lignocellulosic biomass, high dosage of cellulases is still required to hydrolyze lignocellulose to fermentable sugars [8, 9].

To reduce enzyme dosage, various studies have been carried out to develop enzyme recycling technologies [10–14]. As after the enzymatic hydrolysis of lignocellulosic biomass, cellulases remain either bound to the residual substrate or be free in the supernatant [15]. Thus, recycling cellulases could be achieved through recovering cellulases from both the liquid and solid phases [16–18]. Although recycling cellulases from the liquid phase of hydrolysate has been studied extensively, it was reported that the enzymes adsorbed on the solid substrates are the key enzymes that are necessary for effective cellulose hydrolysis [19–21]. This is because the enzymes adsorbed on the solid substrates included the major cellulase degrading enzymes that have been reported to have high binding affinity towards cellulose [15] and have been considered as the rate-limiting enzyme component for the overall cellulose hydrolysis [16]. Different from the bounded cellulases, the free enzymes have been reported to include mainly β-glucosidase activities and hemicellulose activity [15]. In addition, it has been found that removing the “free/unabsorbed” enzymes had only a minor to negligible effect on the yield of cellulose hydrolysis over a range of cellulosic and lignocellulosic substrates [15]. Also, it has been reported there was a linear relationship between the amount of adsorbed enzyme and the hydrolysis yield [17]. Thus, recovering enzymes adsorbed on the solid substrates is theoretically more important. Some studies have investigated recovering the adsorbed enzymes through desorption. Nevertheless, enzyme activity loss is significant and greatly limits its application in a biorefinery [12, 22]. Recycling unhydrolyzed solids with adsorbed enzymes is a promising approach. Previously, we have demonstrated that recycling unhydrolyzed solids recycled 50–60% enzymes and reduced around 40% enzyme loading on ammonia pre-treated biomass [23, 24]. However, recycling unhydrolyzed soldis has not been well studied on other pre-treated biomass in a high solid loading process. As pre-treatment has a great impact on the property of lignocellulosic biomass, it is expected that biomass pre-treated differently has a different enzyme adsorption property and thereby different enzyme recycling potential via recycling unhydrolyzed solids. For example, acid catalyzed pre-treatments eliminated mainly hemicellulose from biomass [25, 26] while alkali pre-treatments can remove lignin [27], which may greatly affect their enzyme adsorption properties and thus affect the effectiveness of enzyme recycling via recycling unhydrolyzed solids.

This work studied the kinetics of cellulose hydrolysis on both dilute acid and dilute alkali pre-treated corn stover, and investigated enzyme adsorption kinetics over the course of cellulose hydrolysis. The role of lignin materials (from dilute acid and dilute alkali pre-treated corn stover) in enzyme adsorption was also studied. Based on the kinetic study, a fast enzyme recycling strategy by recycling unhydrolyzed solids after 24 h hydrolysis was developed in a high solid loading process. Enzyme recycling was performed for 5 rounds and 40%–50% enzyme loading reduction was achieved.

## Materials and methods

### Corn stover and enzymes

Corn stover used in this study was obtained from Lianyungang, Jiangsu Province, China. The particle size of corn stover was reduced by processing corn stover in a Wiley mill with a mesh screen to achieve a particle size of ~ 4 mm. Cellulase (60 mg protein/mL, 118 FPU/mL) and xylanase (25 mg protein/mL, 100,000 U/mL for beechwood xylan) were provided by Qingdao Vland Biotech Inc.

### Corn stover pre-treatments

Dilute acid pre-treatment of corn stover was performed in 2-L pressure reactor (Weihai Chemical Device, Shandong, China). In brief, corn stover was pre-soaked with 1% (w/w) sulfuric acid at 10% (w/w) solid loading. Then, the pre-soaked slurry was transferred into the pressure reactor and pre-treated at 180 °C for 10 min with constant stirring (200 rpm). After pretreatment, the resulting biomass slurry was cooled down, washed with excessive water, oven-dried at 60 °C and stored at 4 °C.

Dilute alkali pre-treatment was conducted in an autoclave. Corn stover was pre-treated at 10% (w/w) solid loading, 2% (w/w) NaOH loading in a 1L flask with total working weight of 500 g at 121 °C for 20 min. After the pretreatment, the pre-treated corn stover was adjusted to neutral pH and washed with excessive water, oven-dried at 60 °C and stored at 4 °C.

### Biomass composition and characterization

The composition analysis of biomass was performed by following the National Renewable Energy Laboratory (NREL, Denver, CO, USA) protocol [28]. Sugar concentrations were measured by high performance liquid chromatography (HPLC) equipped with a refractive index detector and a Biorad Aminex HPX-87H column running at 65 °C. A mobile phase of 5 mM sulfuric acid was used at a flow rate of 0.6 mL/min. Each sample was diluted and filtered through a 0.22 μm Nylon syringe filter before analysis.

### Cellulolytic enzyme lignin (CEL) isolation and characterization

To obtain the enzymatic lignin, the dilute acid/alkali pre-treated corn stover substrates were hydrolyzed using excessive enzymes as described previously [29]. In brief, the pre-treated substrates were enzymatically hydrolyzed at 10% (w/w) solids loading with excessive cellulases and xylanase added with the ratio of 1:1 (protein basis) to make a total enzyme loading of 60 mg protein/g glucan. After 72 h enzymatic hydrolysis, the residue solids were collected by centrifugation and washed with water to remove unbound enzymes. Then the residue solids were further hydrolyzed for another 72 h using the same enzyme loading. The enzymatic hydrolysis step was repeated until no sugar release was detected. The resulting solids were then washed with deionized water, sonicated for 60 min, and further washed with 10 mM sodium acetate and 5 mM calcium acetate (pH 7.5). Protease from Streptomyces griseus (Type XIV, 1 U/mL, Sigma-Aldrich) was then applied to hydrolyze the cellulases adsorbed on solids. Thereafter, protease was deactivated by incubating at 90 °C for 2 h. The obtained residual solids were washed with 1 M NaCl solution and deionized water. Then, the solids (cellulolytic enzyme lignin) were freeze-dried and kept at 4 °C for future use.

The characterization of CEL was performed on Thermo Scientific™ Nicolet™ iS™5 FT-IR spectroscopy with a universal attenuated total reflection (ATR) accessory, which enables samples to be examined directly in the solid state without further preparation. FT-IR spectra were obtained by averaging 16 scans from 4000 to 400 cm^−1^.

### Enzymatic hydrolysis

Enzymatic hydrolysis of dilute acid/alkali pre-treated corn stover were carried out in 250 mL flasks (50 °C, 250 rpm) at 7% (w/w) or 1% (w/w) glucan loading for 96 h. The final weight of the reaction system was 100 g, and 50 mM sodium citrate buffer (pH 4.8) was used as buffer solution. Cellulase and xylanase (Qingdao Vland Biotech Inc., China) used for enzymatic hydrolysis was added with the ratio of 1:1 (protein basis). The total enzyme loadings for dilute acid and dilute alkali pre-treated corn stover were 30 mg protein/g glucan and 40 mg protein/g glucan, respectively. To achieve 70–80% glucan conversion of acid and alkali pre-treated biomass within a reasonable hydrolysis period (72 h), the optimized enzyme loadings were chosen. The reason for selecting a cellulose hydrolysis yield of 70–80% as the goal for effective hydrolysis is that techno-economic models of the overall biomass-to-ethanol process have suggested that achieving complete cellulose hydrolysis generally requires long hydrolysis time and high enzyme loading, adding significantly to the operating costs [30, 31]. Thus, we applied the optimized enzyme loading for enzymatic hydrolysis of acid and alkali pre-treated biomass in this study. After the cellulose hydrolysis, samples were taken at different time points and centrifuged at 10,000 rpm for 10 min to separate the liquid fraction from the solids and the concentrations of sugars in supernatant was determined. Sugar concentrations were measured by high performance liquid chromatography (HPLC) as mentioned above. Yield was calculated by using the following equation:

$$\alpha = \frac{c \times V}{{{\text{a}} \times m}} \times 100\%$$

where *α* is yield (%), a is the coefficient of sugar polymer conversion to monomer (specifically, *a* = 1.111 for glucan conversion to glucose; *a* = 1.136 for xylan conversion to xylose), *m* is the initial mass of glucan or xylan (g), *c* is sugar concentration (g/L), *V* is the volume of liquid hydrolysate (L).

### Enzyme adsorption kinetics and isotherm

To measure adsorption kinetics, the protein content in the supernatant of the enzymatic hydrolysate was determined along the time course of cellulose hydrolysis. The enzyme protein concentration of the supernatant of each sample was measured by a modified Ninhydrin method [32], with bovine serum albumin (BSA) as the standard (0–800 μg/mL). In detail, 200 μL diluted sample was mixed with 100 μL 13.3 g/L NaBH_4_ in a pressure resistant glass tube and incubate at room temperature for 1 h, followed by adding 600 μL 9 M HCl and shaking the tube to remove bubbles. Then the tube was heated at 130 °C for 2 h. After cooling to room temperature, 100 μL hydrolysate was transferred into a 2 mL centrifugation tube and neutralized by 100 μL 5 M NaOH. 200 μL Ninhydrin reagent (Sigma-Aldrich) was added and sample was then heated at 100 °C for 10 min. 1 mL 50% (v/v) ethanol was added after the tube cooling down to room temperature. Finally, 200 μL sample was transferred into a 96 well microplate and the absorbance was read at 570 nm.

To determine the adsorption isotherm, different amount of enzymes (25–2000 μg/mL) were incubated with CEL isolated from dilute acid and dilute alkali pre-treated corn stover in centrifugation tubes at 4 °C for 2 h to reach equilibrium. The total volume of the mixture was 2 mL and the lignin was 1% (w/w) in 50 mM sodium citrate buffer (pH 4.8). After incubation, the slurry was centrifuged at 10,000 rpm for 10 min and the supernatant was collected to measure the non-absorbed protein concentration by ninhydrin method mentioned above.

The adsorption data were fitted by Langmuir adsorption model which was described as follows [33]

$$E_{\text{b}} = \frac{{E_{\text{bm}} K_{\text{a}} E_{\text{f}} }}{{1 + K_{\text{a}} E_{\text{f}} }}$$

where *E*_b_ is the absorbed enzyme (mg/g lignin), *E*_bm_ is the maximum absorb capacity (mg/g lignin), *E*_f_ is the free enzyme concentration in liquid phase (mg/L) and the *K*_a_ is the association constant (L/mg). *K*_p_ = *E*_b_ × *K*_a_ was also calculated as distribution coefficient to indicate the strength of interaction of enzyme and lignin [34].

### Enzyme recycling strategy

The enzyme recycling was performed by recycling the remaining solids after 24 h hydrolysis to next round hydrolysis to ensure that the enzyme absorbed on the unhydrolyzed solids could be recycled at the same time. The 1st round hydrolysis of acid and alkali pre-treated CS was performed at 7% glucan solid loading with enzyme dosage of 30 and 40 mg protein/g glucan, respectively. The cellulase and xylanase were added with the ratio of 1:1 (protein basis). The enzymatic hydrolysis was performed at 50 °C, 250 rpm. After 24 h, the unhydrolyzed solid was separated by centrifuge at 10,000 rpm for 10 min. Then, the recycled solids with enzyme adsorbed were mixed with fresh biomass and fresh enzymes for the next round of hydrolysis.

## Results and discussion

### Biomass composition comparison of acid and alkali pre-treated corn stover

To study the effect of pre-treatment on enzyme adsorption, dilute acid and alkali pre-treatments were carried out under optimized conditions to achieve efficient cellulose hydrolysis. The compositions of corn stover before and after pre-treatments are shown in Table 1. Dilute acid pre-treatment removed xylan and increased glucan and lignin contents to 50.8% and 40.1%, respectively. This is expected as previous studies have reported that the inter linkages present in hemicellulose are liable to acid catalysis, leading to the solubilization of hemicellulose into monomeric and oligomeric sugars [35]. Compared with hemicellulose, the lignin in biomass is more recalcitrant [35]. Although partial β-aryl ether units of lignin were cleaved, the majority of lignin was still present in the pre-treated lignocellulosic substrates [36, 37].

**Table 1:** Composition of untreated, dilute alkali treated and dilute acid treated corn stover

Substrate	Glucan (%)	Xylan (%)	Lignin and ash (%)
Untreated	37.2 ± 0.6	19.2 ± 0.1	23.3 ± 0.5
Dilute acid treated	50.8 ± 2.3	n.d.	40.1 ± 0.5
Dilute alkali treated	54.6 ± 0.6	21.8 ± 0.2	10.9 ± 0.7

*n.d.* not detected

Compared with acid pre-treated corn stover, the alkali pre-treated corn stover contained similar glucan content (54.6%), higher xylan content (21.8%), but much lower lignin content (10.9%). It is well known that alkaline-based pre-treatments are efficient in lignin removal [36, 38]. Due to the alkaline hydrolysis, the lignin structure was disrupted and the intermolecular ester bonds that crosslink hemicelluloses and other components were saponified [39–41], resulting in an increased cellulose accessibility and reduced lignin content [42]. The composition differences of acid and alkali pre-treated corn stover render them excellent substrates for comparison studies of enzyme hydrolysis, enzyme adsorption, and enzyme recycle via recycling unhydrolyzed solids.

### Enzymatic hydrolysis of pre-treated corn stover

**Fig. 1:**
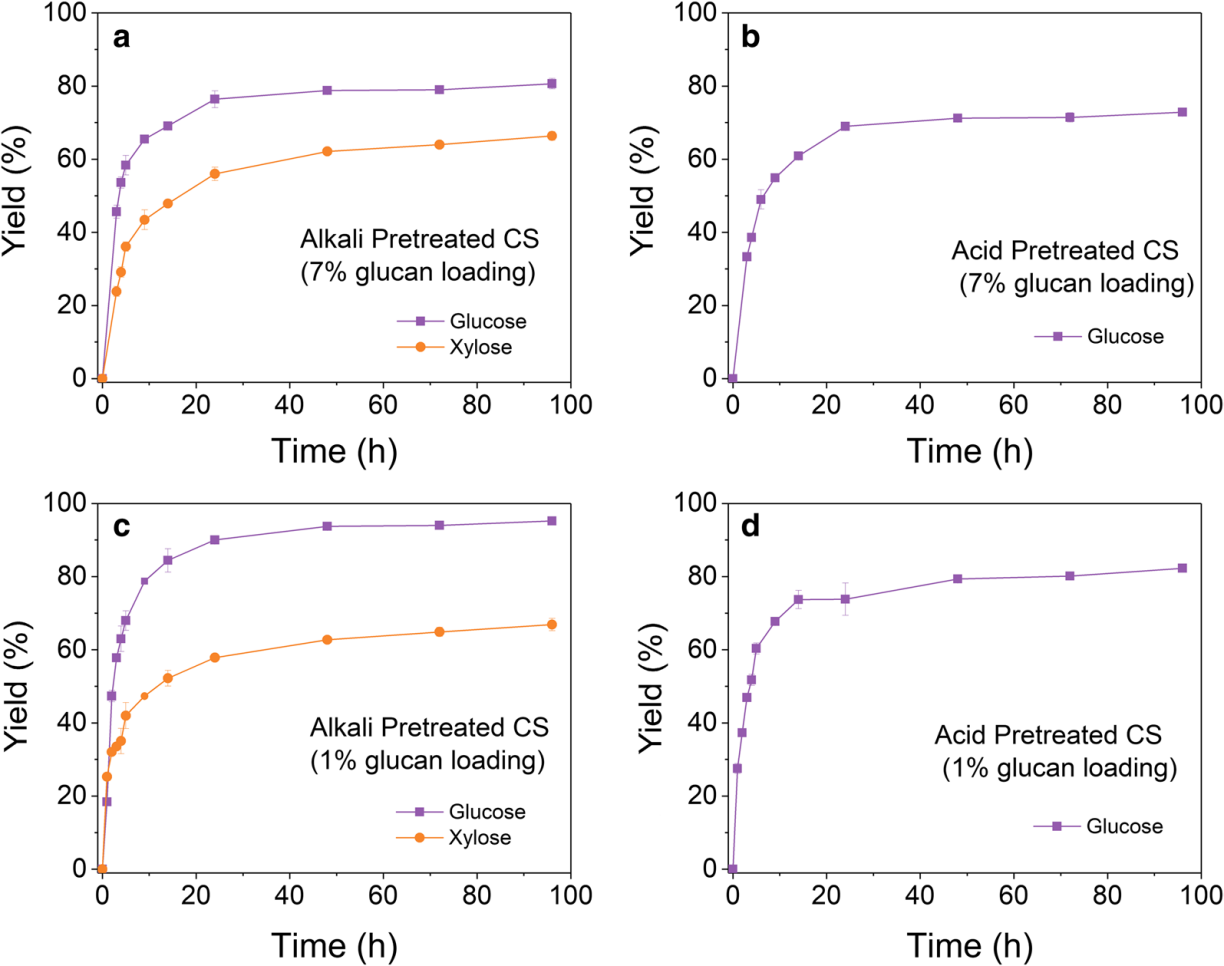
Enzymatic hydrolysis of alkali (**a**, **c**) and acid (**b**, **d**) pre-treated corn stover at 7% (**a**, **b**) and 1% (**c**, **d**) glucan loading. Enzymatic hydrolysis was performed in triplicate. Based on one-way ANOVA, there is significant difference in enzymatic hydrolysis between alkali and acid pre-treated corn stover (*p *= 0.036)

As acid and alkali pre-treated biomass have substantial differences in composition, it is expected that their structural differences have an impact on cellulose hydrolysis and enzyme adsorption. As shown in Fig. 1a, for the alkali pre-treated biomass, glucan and xylan were converted rapidly to glucose and xylose at the beginning of enzymatic hydrolysis at 7% glucan loading and the hydrolysis rates slowed down after 24 h. The glucose and xylose concentrations reached 68.3 g/L and 20.4 g/L at 24 h, representing about 76.4% glucan conversion and 56.0% xylan conversion, respectively. At 96 h, the glucose and xylose concentrations were 71.5 g/L and 24.2 g/L, representing about 80.7% glucan conversion and 66.4% xylan conversion, respectively. It is clear that the initial enzymatic hydrolysis within 24 h accounts for most of the hydrolysis yield within 96 h. Following the similar trend, enzymatic hydrolysis of dilute acid pre-treated corn stover at 7% glucan loading released 62.4 g/L at 24 h and only further released additional 3.5 g/L glucose in the following 72 h (Fig. 1b).

One might suspect that the hydrolysis rate reduction is due to the accumulation of high sugar concentration at high solid loading (7% glucan loading), which inhibit enzyme activities. This might be a cause. Nevertheless, the enzymatic hydrolysis rates reduction after initial hours was also observed when enzymatic hydrolysis was performed at a low solid loading (1% glucan loading, Fig. 1c, d). Therefore, it seems the pre-treated corn stover materials also contained a portion of biomass that is still relatively recalcitrant to hydrolyze, which might be another cause of hydrolysis rate reduction. In addition, it is also possible that the partial lignin in pre-treated corn stover will interact with cellulose and prevent the enzymes from reaching the active sites of cellulose, leading to the decreased hydrolysis rate.

To recycle adsorbed enzymes through recycling unhydrolyzed solids, it would be wise to end enzymatic hydrolysis at 24 h, if the amount of enzymes adsorbed on unhydrolyzed solid substrates is significant, so that the slow hydrolysis period could be avoided. This approach shortens the enzymatic hydrolysis time and the unhydrolyzed biomass can be further hydrolyzed in the subsequent hydrolysis cycles. In addition, enzyme activities could be largely preserved as the hydrolysis time is short (extended hydrolysis time would lose additional enzyme activities).

### Enzyme adsorption during enzymatic hydrolysis

**Fig. 2:**
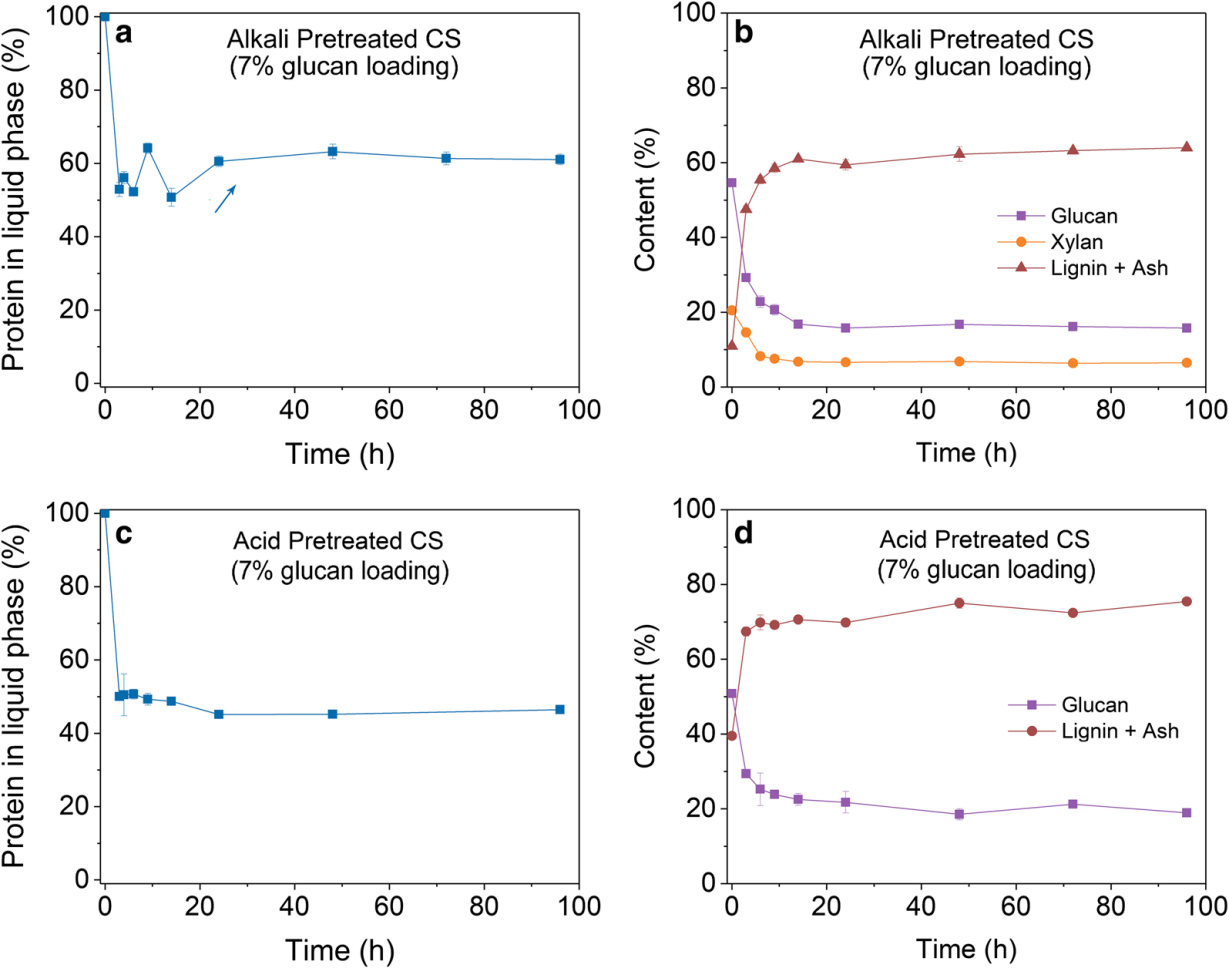
Enzyme adsorption profile and biomass composition change during enzymatic hydrolysis of dilute alkali and dilute acid pre-treated corn stover at 7% glucan loading. **a** Percentage of enzyme protein in liquid phase (un-adsorbed on biomass solids) during enzymatic hydrolysis of dilute alkali pre-treated corn stover; **b** biomass composition change during enzymatic hydrolysis of dilute alkali pre-treated corn stover; **c** percentage of enzyme protein in liquid phase (un-adsorbed on biomass solids) during enzymatic hydrolysis of dilute acid pre-treated corn stover; **d** biomass composition change during enzymatic hydrolysis of dilute acid pre-treated corn stover

As discussed in the introduction section, recovering adsorbed enzymes via recycling unhydrolyzed solids has the potential to be applied in industrial conditions. To realize high enzyme recycling efficiency, understanding enzyme adsorption behaviors on the pre-treated substrates during the course of cellulose hydrolysis is essential. Thus, we determined the enzyme adsorption profiles during enzymatic hydrolysis of dilute alkali and dilute acid pre-treated corn stover materials at 7% glucan loading. During hydrolysis of alkali pre-treated corn stover, enzyme protein concentration in liquid phase decreased rapidly at the beginning to 53% and then fluctuated between 52 and 61% in the first 24 h (Fig. 2a), indicating that around 39%–48% enzymes were adsorbed on the solid substrates in the first 24 h. After 24 h, the enzyme absorption maintained at around 40%. Through composition analysis of unhydrolyzed solids during enzymatic hydrolysis, it is clearly that the composition of solid substrate changed dramatically during the first 24 h with glucan content decreased from 54.6% to around 16% and lignin-ash content increased from 10.9% to around 60% (Fig. 2b). It was expected that with hydrolysis of glucan (the substrate of cellulases) a substantial percentage of cellulases would desorb from solid biomass [43]. However, less than 10% increase of unbound enzymes in liquid phase was observed. Hydrolysis may open up significant amount of enzyme adsorption sites on lignin, and with the progress of hydrolysis more enzymes might have adsorbed to lignin, which may explain small change in adsorbed enzyme percentage during enzymatic hydrolysis. Similar enzyme adsorption profile was observed for enzymatic hydrolysis of dilute acid pre-treated corn stover (Fig. 2c). Around 50% of total enzyme protein was adsorbed on unhydrolyzed solids at the beginning of hydrolysis and this number went up to 55% at 24 h with lignin-ash content in unhydrolyzed solids increased to 70% (Fig. 2d). Overall, it seems lignin played a significant role in enzyme adsorption when most of the glucan got hydrolyzed.

More enzymes absorbed on acid pre-treated biomass residuals compared to alkali pre-treated biomass. This could be caused by the differences in composition and structure of the substrates and the hydrolysis yield. Previous studies reported that the properties of lignin affected the cellulase adsorption profile on pre-treated substrates [44, 45]. As alkali pre-treated corn stover contained less amount of lignin while acid pre-treated corn stover contained much higher lignin content (Table 1), it is likely that lignin is the major factor led to more enzymes bound to acid pre-treated corn stover. Moreover, acid pre-treatment and alkali pre-treatment may modify the lignin in a different way and thus result in different enzyme absorption property of the two kinds of lignin.

### Enzyme adsorption comparison on lignin materials derived from acid and alkali pre-treated corn stover

**Fig. 3:**
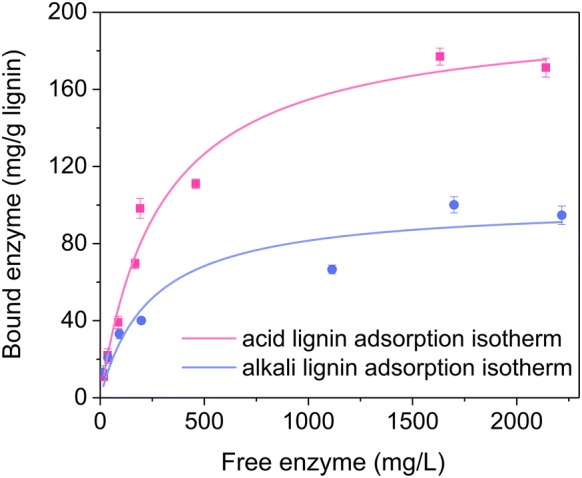
Adsorption isotherms of cellulase on cellulolytic enzyme lignin materials derived from acid and alkali pre-treated corn stover. The adsorption experiments were performed by mixing CEL (solid loading at 1%), enzyme (cellulase loading at 25–2000 μg/mL) and 50 mM sodium citrate buffer (pH 4.8) at a total volume of 2 mL, followed by incubation at 4 °C for 60 min to reach equilibrium. Error bars represent standard errors

To further understand the role of lignin materials in enzyme adsorption, we enzymatically hydrolyzed dilute acid pre-treated and dilute alkali pre-treated corn stover excessively to remove all the carbohydrates that are hydrolyzable by the enzyme cocktail applied. The obtained cellulolytic enzyme lignin (CEL) materials were used to investigate their enzyme adsorption kinetics (Fig. 3), and the Langmuir adsorption model was used to fit the data (Table 2). It was found that CEL isolated from dilute alkali pre-treated corn stover (CEL-alkali-CS) had a maximum enzyme adsorption capacity (*E*_bm_) of 100.9 mg protein/g CEL, and the CEL from dilute acid pre-treated corn stover (CEL-acid-CS) had a much higher *E*_bm_ of 199.0 mg protein/g CEL. The association constant for CEL-alkali-CS and CEL-acid-CS was 4.2 mL/mg and 3.5 mL/mg, respectively. The distribution coefficient (*K*_p_) was also calculated to characterize the interaction between enzyme and CEL. *K*_p_ of CEL-acid-CS was 696.4 mL/g, which was higher than 423.8 for CEL-alkali-CS, indicating that lignin isolated from dilute acid pre-treated corn stover had a higher enzyme adsorption capability than CEL-alkali-CS. Therefore, the enzyme adsorption difference in enzymatic hydrolysis of acid pre-treated CS and alkali pre-treated CS (Fig. 2) is also due to the adsorption property difference of lignin.

**Table 2:** Langmuir adsorption isotherm parameters for cellulase adsorbing onto cellulolytic enzyme lignin (CEL) materials

Lignin	*E*_bm_ (mg/g)	*K*_a_ (mL/mg)	*K*_p_ (mL/g)	Adj. *R*-square
CEL(alkali-CS)^a^	100.9	4.2	423.8	0.975
CEL(acid-CS)^b^	199.0	3.5	696.4	0.905

^a^CEL(alkali-CS): cellulolytic enzyme lignin derived from alkali pre-treated corn stover containing 7.5% glucan, 2.3% xylan and 75.3% lignin and ash

^b^CEL(acid-CS): cellulolytic enzyme lignin derived from acid pre-treated corn stover containing 2.7% glucan, 0.0% xylan and 86.6% lignin and ash

**Fig. 4:**
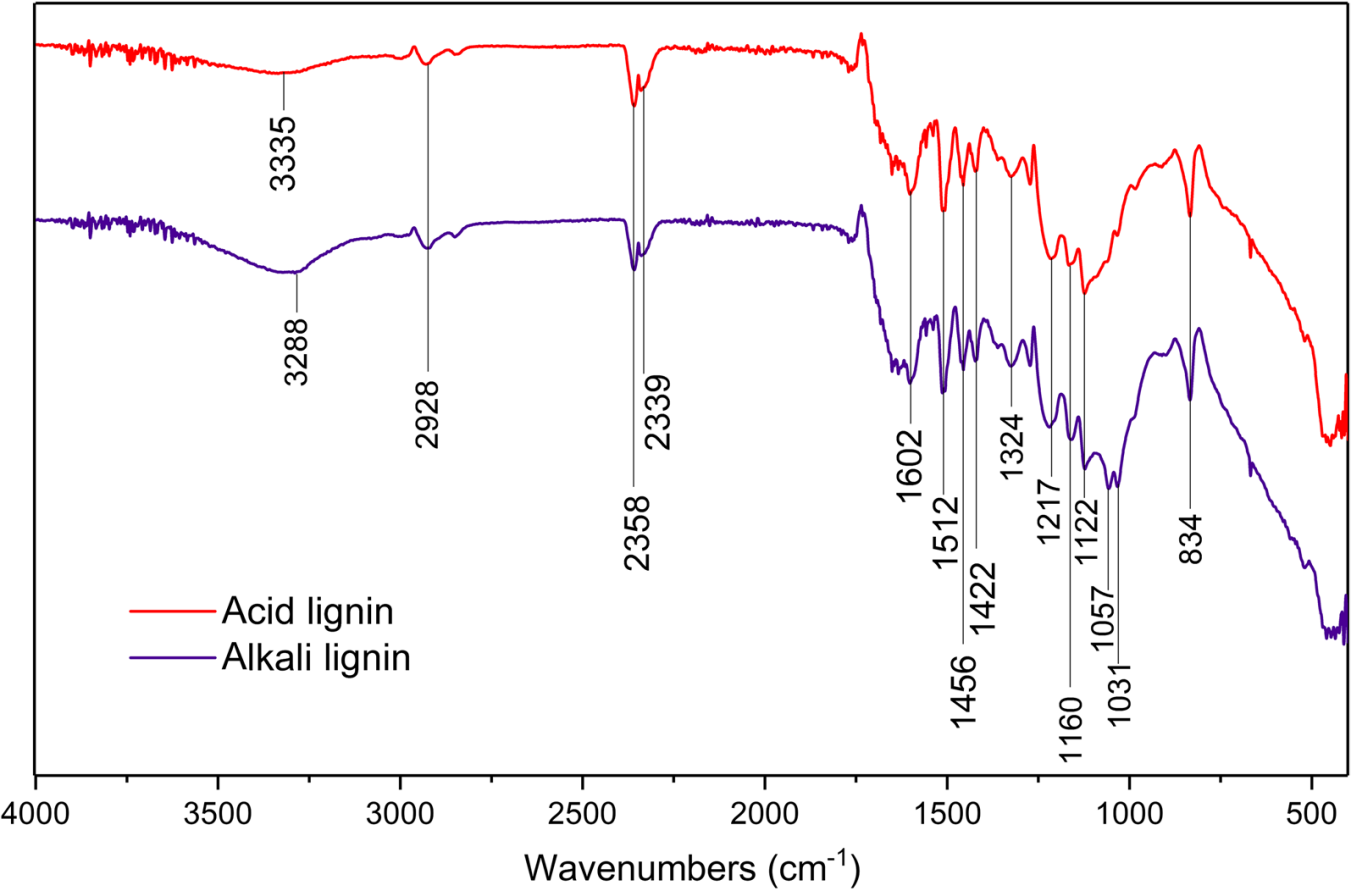
FT-IR spectra of cellulolytic enzyme lignin derived from acid and alkali pre-treated corn stover

To investigate the cause for the difference in enzyme adsorption property between CEL-alkali-CS and CEL-acid-CS, we further analyzed the CELs by FT-IR (Fig. 4). The CEL-alkali-CS and CEL-acid-CS showed very similar functionalities (Fig. 4 and Table 3), except that CEL-alkali-CS’s broad band at 3300–3400 cm^−1^ was stronger than that of CEL-acid-CS, suggesting that CEL-alkali-CS contained more hydroxyl group, which could increase its hydrophilicity. This might be a cause for lower enzyme adsorption capacity of CEL-alkali-CS since hydrophobic interactions play an important role in enzyme adsorption to biomass [46, 47].

**Table 3:** Signal assignment in FT-IR spectra of lignin derived from acid and alkali pretreamtent

Wavenumbers (cm^−1^)	Attribute
3288, 3335	Aromatic or aliphatic O–H stretching vibration
2928	–CH_2_, –CH_3_ groups C–H vibration
2358, 2339	Carboxylic group C–O stretching
1602	Benzene rings C–C stretching vibration
1512	Benzene rings C–C stretching vibration
1456	Methyl and methylene C–H deformation vibration
1422	Benzene rings C–C stretching vibration
1324	Lignin syringyl unit C–H and C–O vibration
1271	Aromatic methyl ether bridges
1057	Aromatic methyl ether bridges
1160	Aromatic C–H deformation vibration of syringyl unit rings and aromatic C–H in plane deformation of guaicyl and syringyl units of lignin
1122	
1031	
834	Lignin syringyl unit C–H and C–O vibration

**Fig. 5:**
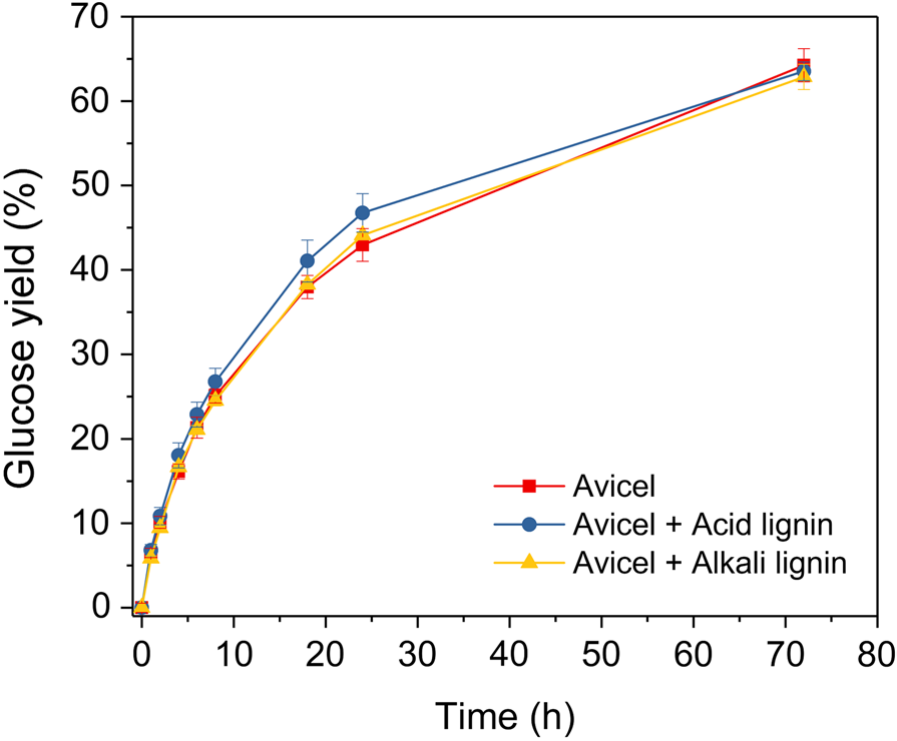
Effect of cellulolytic enzyme lignin on enzymatic hydrolysis of Avicel at 2% Avicel loading and 1% lignin loading (the experiment was performed at 50 °C in 50 mM sodium citrate buffer (pH 4.8) at cellulase loading of 5 mg protein/g glucan)

Through the above studies, it can conclude that it is feasible to end enzymatic hydrolysis at 24 h and recycle enzymes through recycling unhydrolyzed solids as there were significant amount of enzymes adsorbed on solids (mostly lignin). As lignin plays an important role in enzyme adsorption and recycling in this approach, it is essential to know how the lignin affects enzymatic hydrolysis. Therefore, we further examined the effects of CEL-alkali-CS and CEL-acid-CS on enzymatic hydrolysis of Avicel cellulose. Surprisingly, even a low enzyme loading (5 mg protein/g glucan) was applied, no significant effect of lignin was observed. The hydrolysis with addition of CEL-alkali-CS or CEL-acid-CS showed almost the same sugar yield pattern as the one without any lignin (Fig. 5). It seems these two lignin materials did not have significant inhibitory effect. As reported earlier, the affinity of cellulose to cellulases is higher compared to lignin [48], and thus the enzymes recycled by adsorbing to lignin could potentially “jump” to cellulose when cellulose is available. This is because enzyme adsorption on pre-treated biomass is dependent upon multiple intrinsic properties of the proteins and the composition of pre-treated biomass. All of these interactions occur in a competitive way, until an equilibrium driven by the enzyme adsorption for the various pre-treated biomass has been reached. Among the components of pre-treated biomass, lignin in the pre-treated biomass has been considered as an essential factor that affect enzyme adsorption on pre-treated biomass. Some studies suggest that lignin could cause non-productive binding through binding cellulases through hydrophobic interactions, electrostatic interactions and hydrogen bonding forces [49, 50]. However, the amount of enzyme adsorption on lignin did not illustrate the types of enzyme adsorbed on lignin. Recently, it has been reported that β-glucosidase in commercial cellulases mixture adsorbed preferably on lignin in the presence of cellulose and remained active, while the exocellulases preferably on cellulose [51]. This can be considered as the potential reason for why lignin has high cellulase adsorption capacity but no obvious effect on the hydrolysis of avicel when supplemented with lignin. Specifically, despite the high adsorption capacity of lignin towards cellulase, the essential cellulose-degrading enzyme such as exocellulases could adsorb initially on cellulose in the presence of lignin at the beginning stage of hydrolysis. With increase of cellulose conversion, the adsorbed cellulase on cellulose might return to solution and part of the enzymes might get reabsorbed on lignin, facilitating the enzyme recycling process through recycling unhydrolyzed biomass.

In addition, one might suspect that the difference (about 10%) of sugar yield between alkali and acid pre-treated CS (Fig. 1) indicated that lignin might play a role in decreasing the efficiency of hydrolysis, which seems not consistent with the result shown in Fig. 5. However, such phenomena may be related with the difference in the accessibility of cellulose due to different level of lignin content in the pre-treated substrates. In the pre-treated substrates, lignin interacts with pre-treated substrates, limiting cellulose accessibility to cellulases [24]. Thus, higher lignin content of acid pre-treated substrates indicates more lignin may interact with cellulose, resulting in lower cellulose accessibility and lower sugar yield compared with that of alkali pre-treated substrate. Differently, during the hydrolysis of avicel in the presence of supplemented lignin, both acid and alkali lignin did not form such interaction with cellulose so that cellulose is still accessible for enzymatic hydrolysis, which might result in their negligible effect on enzymatic hydrolysis of avicel.

### Enzyme recycling via recycling unhydrolyzed solids

**Fig. 6:**
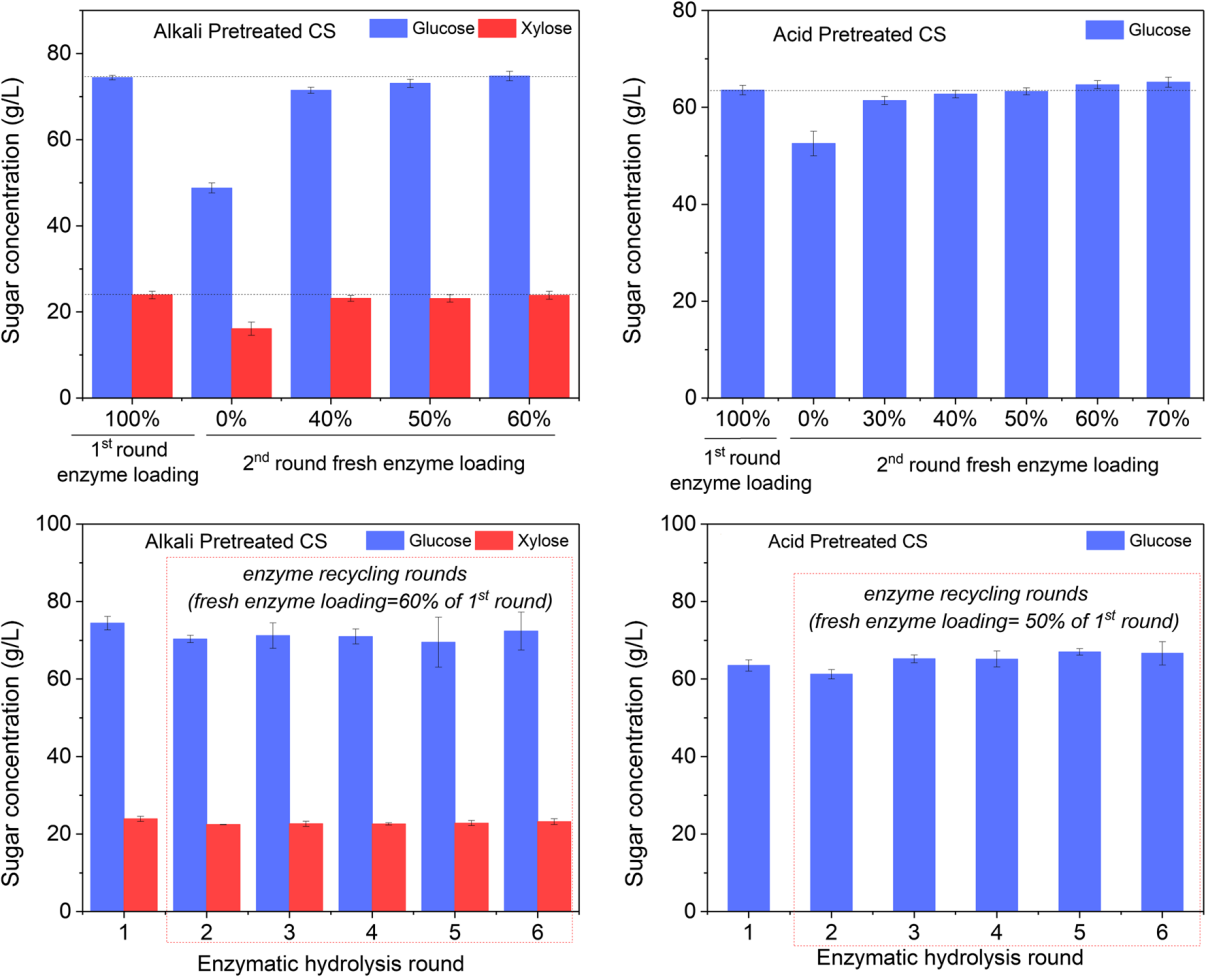
Performance of enzymatic hydrolysis using enzymes recycled via recycling unhydrolyzed solids. Enzymatic hydrolysis was carried out on dilute alkali pre-treated corn stover or on dilute acid pre-treated corn stover for 24 h (1st round). The resulting unhydrolyzed solids were recycled to the subsequent round of enzymatic hydrolysis. Enzymatic hydrolysis was performed for 24 h for all rounds. **a** Effect of fresh enzyme loading in the second round enzymatic hydrolysis of dilute alkali pre-treated corn stover on glucose yield; **b** effect of fresh enzyme loading in the second round enzymatic hydrolysis of dilute acid pre-treated corn stover on glucose yield; **c** five rounds of enzyme recycling for enzymatic hydrolysis of dilute alkali pre-treated corn stover using 60% fresh enzyme loading for round 2–6; **d** five rounds of enzyme recycling for enzymatic hydrolysis of dilute acid pre-treated corn stover using 50% fresh enzyme loading for round 2–6

As there is substantial amount of enzymes adsorbed on unhydrolyzed biomass during enzymatic hydrolysis, and 24 h hydrolysis already hydrolyzed most of the hydrolysable biomass, it would be wise to perform enzymatic hydrolysis for only 24 h and recycle the unhydrolyzed solids to the next round hydrolysis so that enzymes can be recycled and enzymatic hydrolysis time can be reduced. The recycled unhydrolyzed solids would be further hydrolyzed in the subsequent hydrolysis rounds. To verify the feasibility of this approach, we enzymatically hydrolyzed acid pre-treated and alkali pre-treated corn stover for 24 h (1st round hydrolysis) and recycled the unhydrolyzed solids to the next round hydrolysis (2nd round hydrolysis), where the same amount of fresh biomass and water/buffer as the first round were added and different amount of fresh enzymes was dosed. As shown in Fig. 6a, b, without supplementation of fresh enzymes, the second round hydrolysis of dilute alkali pre-treated corn stover and dilute acid pre-treated corn stover reached 65.6% and 82.5% of the glucose concentration achieved by the first round, respectively. The glucose concentration for the second round hydrolysis increased with the increase of enzyme loading. On dilute alkali pre-treated corn stover, around 60% fresh enzyme loading (assuming the enzyme loading for the first round hydrolysis is 100%) is needed for the second round hydrolysis to reach the statistically similar glucose concentration as the first round (one-way ANOVA test, *p* =0.973 > 0.05) (Fig. 6a), while on the dilute acid pre-treated corn stover 50% fresh enzyme loading is sufficient (Fig. 6b). This is consistent with our adsorption study, which showed that after 24 h enzymatic hydrolysis of alkali and acid pre-treated corn stover there were 40% and 55% of the enzymes, respectively, adsorbed on unhydrolyzed solids (Fig. 2), which were theoretically recycled to the second round hydrolysis and thus resulted in corresponding reductions in fresh enzyme loading. Therefore, recycling enzymes via recycling unhydrolyzed solids is viable on dilute acid and dilute alkali pre-treated corn stover.

**Fig. 7:**
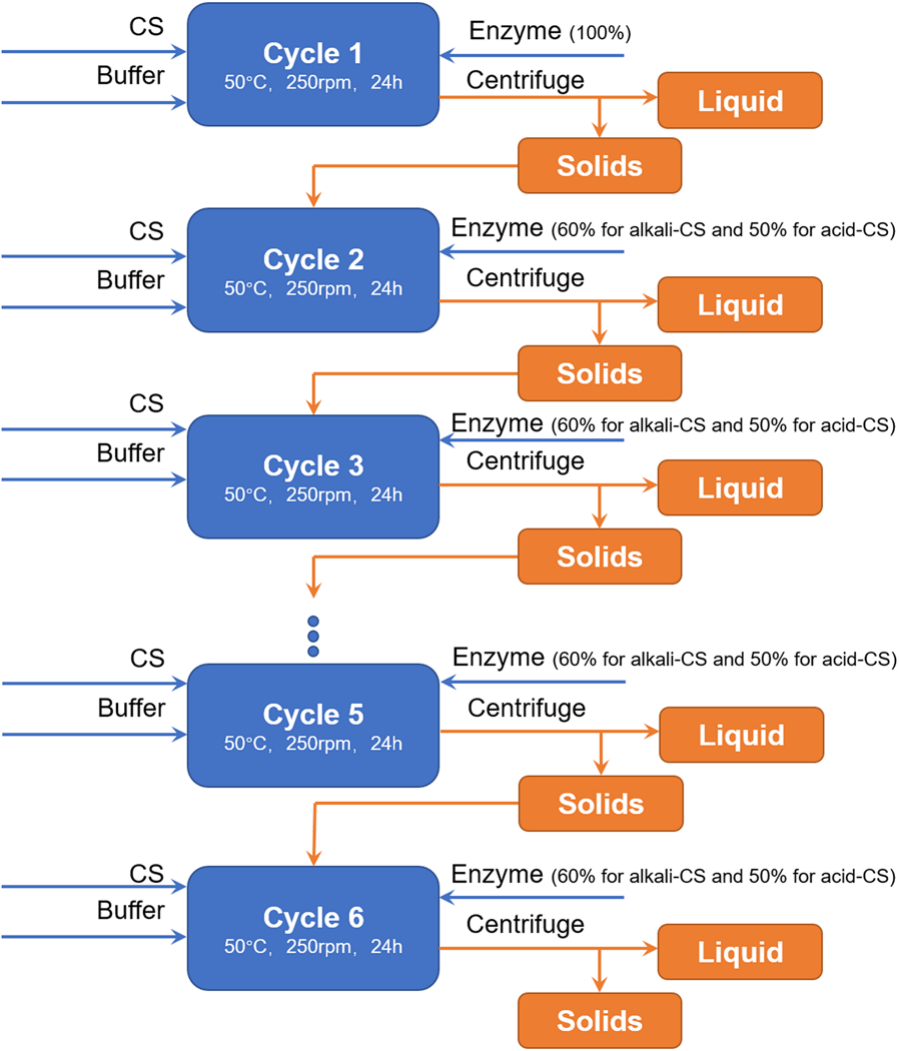
Flow chart of enzyme recycling strategy. In each cycle, fresh dilute acid and alkali pre-treated corn stover were added at 7% glucan loading. The fresh enzyme loading for 1st round were 30 and 40 mg protein/g glucan for alkali and acid pre-treated corn stover, respectively. From 2nd to 6th round, the added fresh enzyme loadings were reduced to 60% and 50% of 1st round for alkali and acid pre-treated corn stover, respectively

We further tested 5 rounds 24 h hydrolysis and enzyme recycling. Unhydrolyzed solids were kept recycled after 24 h hydrolysis for five sequential rounds (Fig. 7). On dilute alkali pre-treated corn stover, enzymatic hydrolysis rounds 2–6 loaded 60% of the fresh enzymes loaded for the first round, and on dilute acid pre-treated corn stover, 50% of the fresh enzymes was loaded for rounds 2–6. As shown in Fig. 6c, d, the sugar concentrations for enzymatic hydrolysis rounds 2–6 reached statistically similar levels as the first round (one-way ANOVA test, *p* = 0.697 > 0.05), which verified the efficiency of enzyme recycling via this approach. This approach reduced 40% and 50% enzyme loading for dilute alkali pre-treated corn stover and for dilute acid pre-treated corn stover, respectively.

## Conclusions

This work studied both enzymatic hydrolysis kinetics and enzyme adsorption kinetics on pre-treated corn stover, and developed enzyme recycling strategy based on kinetic studies. It was found that most of the hydrolysable biomass was hydrolyzed in the first 24 h and about 40% and 55% of the enzymes were adsorbed on unhydrolyzed solids for dilute alkali pre-treated corn stover and dilute acid pre-treated corn stover, respectively. Lignin played a significant role in such adsorption. Recycling enzymes via recycling unhydrolyzed solids reduced 40% and 50% of the enzyme loadings for hydrolysis of dilute alkali-CS and for hydrolysis of dilute acid-CS, respectively.

## Abbreviations

CS: corn stover
CEL: cellulolytic enzyme lignin
HPLC: high performance liquid chromatography
BSA: bovine serum albumin
ATR: attenuated total reflection
