# Correction to: Developing fast enzyme recycling strategy through elucidating enzyme adsorption kinetics on alkali and acid pretreated corn stover

**DOI:** 10.1186/s13068-018-1322-6

**Published:** 2018-12-10

**Authors:** Ye Yuan, Rui Zhai, Ying Li, Xiangxue Chen, Mingjie Jin

**Affiliations:** School of Environmental and Biological Engineering, Nanjing University of Science and Technology, 200 Xiaolingwei Street, Nanjing, 210094 China

## Correction to: Biotechnol Biofuels (2018) 11:316 10.1186/s13068-018-1315-5

Following publication of the original article [1], the authors wish to update the information under the heading “Enzyme adsorption comparison on lignin materials derived from acid and alkali pre-treated corn stover”. The corrected text is as follows: 

Enzyme adsorption comparison on lignin materials derived from acid and alkali pre-treated corn stover.

To further understand the role of lignin materials in enzyme adsorption, we enzymatically hydrolyzed dilute acid pre-treated and dilute alkali pre-treated corn stover excessively to remove all the carbohydrates that are hydrolyzable by the enzyme cocktail applied. The obtained cellulolytic enzyme lignin (CEL) materials were used to investigate their enzyme adsorption kinetics (Fig. 3), and the Langmuir adsorption model was used to fit the data (Table 2). It was found that CEL isolated from dilute alkali pre-treated corn stover (CEL-alkali-CS) had a maximum enzyme adsorption capacity (Ebm) of 10.09 mg protein/g CEL, and the CEL from dilute acid pre-treated corn stover (CEL-acid-CS) had a much higher Ebm of 19.90 mg protein/g CEL. The association constant for CEL-alkali-CS and CEL-acid-CS was 4.2 mL/mg and 3.5 mL/mg, respectively. The distribution coefficient (Kp) was also calculated to characterize the interaction between enzyme and CEL. Kp of CEL-acid-CS was 69.64 mL/g, which was higher than 42.38 for CEL-alkali-CS, indicating that lignin isolated from dilute acid pre-treated corn stover had a higher enzyme adsorption capability than CEL-alkali-CS. Therefore, the enzyme adsorption difference in enzymatic hydrolysis of acid pre-treated CS and alkali pre-treated CS (Fig. 2) is also due to the adsorption property difference of lignin.

Further to this, the authors reported an error in Fig. 3 and Table 2.

The corrected Fig. 3 and Table 2 are provided here.

**Fig. 3:**
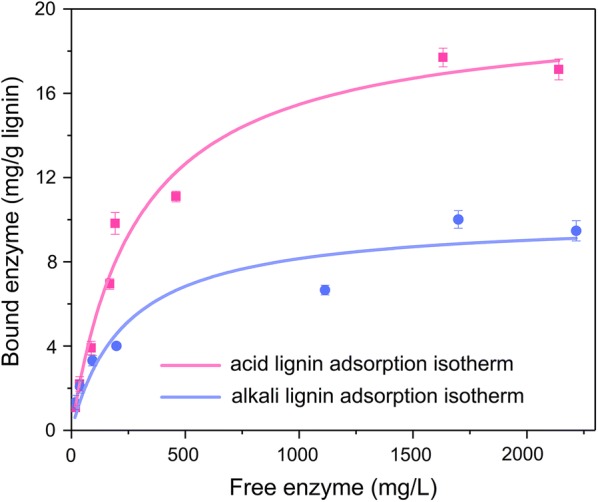
Adsorption isotherms of cellulase on cellulolytic enzyme lignin materials derived from acid and alkali pre-treated corn stover. The adsorption experiments were performed by mixing CEL (solid loading at 1%), enzyme (cellulase loading at 25–2000 μg/mL) and 50 mM sodium citrate buffer (pH 4.8) at a total volume of 2 mL, followed by incubation at 4 °C for 60 min to reach equilibrium. Error bars represent standard errors

**Table 2:** Langmuir adsorption isotherm parameters for cellulase adsorbing onto cellulolytic enzyme lignin (CEL) materials

Lignin	*E*_bm_ (mg/g)	*K*_a_ (mL/mg)	*K*_p_ (mL/g)	Adj. *R*-square
CEL (alkali-CS)^a^	10.09	4.2	42.38	0.975
CEL (acid-CS)^b^	19.90	3.5	69.64	0.905

^a^CEL (alkali-CS): cellulolytic enzyme lignin derived from alkali pre-treated corn stover containing 7.5% glucan, 2.3% xylan and 75.3% lignin and ash

^b^CEL (acid-CS): cellulolytic enzyme lignin derived from acid pre-treated corn stover containing 2.7% glucan, 0.0% xylan and 86.6% lignin and ash
